# Tau Passive Immunotherapy in Mutant P301L Mice: Antibody Affinity versus Specificity

**DOI:** 10.1371/journal.pone.0062402

**Published:** 2013-04-29

**Authors:** Cristina d’Abramo, Christopher M. Acker, Heidy T. Jimenez, Peter Davies

**Affiliations:** 1 Litwin-Zucker Center for Research in Alzheimer’s Disease, Feinstein Institute for Medical Research, North Shore LIJ Health System, Manhasset, New York, United States of America; 2 Department of Pathology, Albert Einstein College of Medicine, Bronx, New York, United States of America; New York University, United States of America

## Abstract

The use of antibodies to treat neurodegenerative diseases has undergone rapid development in the past decade. To date, immunotherapeutic approaches to Alzheimer’s disease have mostly targeted amyloid beta as it is a secreted protein that can be found in plasma and CSF and is consequently accessible to circulating antibodies. Few recent publications have suggested the utility of treatment of tau pathology with monoclonal antibodies to tau. Our laboratory has begun a systematic study of different classes of tau monoclonal antibodies using mutant P301L mice. Three or seven months old mutant tau mice were inoculated weekly with tau monoclonal antibodies at a dose of 10 mg/Kg, until seven or ten months of age were reached respectively. Our data strongly support the notion that in P301L animals treated with MC1, a conformational monoclonal antibody specific for PHF-tau, the rate of development of tau pathology is effectively reduced, while injecting DA31, a high affinity tau sequence antibody, does not exert such benefit. MC1 appears superior to DA31 in overall effects, suggesting that specificity is more important than affinity in therapeutic applications. Unfortunately the survival rate of the P301L treated mice was not improved when immunizing either with MC1 or PHF1, a high affinity phospho-tau antibody previously reported to be efficacious in reducing pathological tau. These data demonstrate that passive immunotherapy in mutant tau models may be efficacious in reducing the development of tau pathology, but a great deal of work remains to be done to carefully select the tau epitopes to target.

## Introduction

Passive immunization with appropriate amyloid beta (Aß) antibodies has been shown to reduce extracellular amyloid deposition in hAPP transgenic mice [1]–[4] [1], [2], [3], [4], and numerous humanized monoclonal antibodies to various Aß epitopes are making their way into clinical trials [5]. The last few years has seen major developments in the tau field that seem likely to have a significant impact on the development of strategies to target insoluble tau aggregates. The idea that tau pathology can diffuse from cell to cell in a prion-like fashion has been shown by different laboratories [6]–[10]. Additional publications seem to confirm the spreading of pathological tau in certain transgenic mouse models [11], [12], again implying the existence of an extracellular tau species that is important in the development of the disease. These studies together with more recent data showing that tau is actively released from cultured cells [13], [14] suggest that, even under normal conditions, a significant amount of tau is present in the extracellular space. In this context, assuming that tau is at least in part an extracellular protein, efforts to target tau pathology with antibodies appear to be a reasonable exercise.

Recent studies from different laboratories have strongly suggested that immunotherapy can be an effective means of preventing the development of tau accumulation [15]–[19]. Our initial approach to passive immunotherapy was to attempt to classify the available tau monoclonal antibodies into groups, based on specificity for tau pathology relative to reactivity with normal tau. The assumption was that the nature of the extracellular tau responsible for the spread of tau pathology was distinct from the normal tau form present in CSF of healthy individuals. In the present study, we have selected three tau monoclonal antibodies that were produced and characterized in our laboratory: MC1, DA31 and PHF1 [20], [21]. DA31 is a pan-tau antibody that maps in the amino acid region 150–190 of tau; PHF1 detects the pSer396/404 tau epitope present on both normal adult brain tau and PHF-tau, while MC1 recognizes a very specific early pathological tau conformation produced by the intramolecular association between the extreme N-terminus and the third microtubule repeat domain of tau. P301L mice at different ages were immunized with the tau monoclonal antibodies previously described, in an attempt to reduce insoluble tau aggregates and increase their survival rate. Here we show that, in tau mutant P301L mice, monoclonal antibody specificity rather than affinity plays a crucial role in clearing tau pathology.

## Materials and Methods

### Ethics Statement

Animals were used in full compliance with the National Institutes of Health/Institutional Animal Care and Use Committee guidelines. The protocol was approved by the Institutional Animal Care and Use Committee of The Feinstein Institute, under protocol # 2007-029.

### Mice

Cohorts (N = 15 per group) of female JNPL3 were used in our study (Taconic Farms). JNPL3 mice express 0N4R human tau with the P301L mutation that causes frontotemporal dementia in humans, under the mouse prion promoter. These mice develop neurofibrillary tangles (NFT) and in later stage progressive deterioration of the motor function. The main advantage of this model is the relatively early onset of the pathology.

### Antibodies

P301L mice were treated for 4 months with weekly intraperitoneal (IP) injections of purified mouse monoclonal antibodies or saline. MC1, DA31 and PHF1 were used at a dose of 10 mg/Kg. The dose of monoclonal antibody was chosen based on the use of monoclonal antibodies in humans, which is usually in the range of 1–10 mg/Kg. The duration of the treatment was established by preliminary experiments in which the extent of pathology was monitored in untreated animals of different ages. Although there is considerable variability in the rate of development of tau pathology in the P301L mice, power calculations suggested that a 40% reduction in pathology would be detectable over 4 months with 15 animals per group.

**Figure 1:**
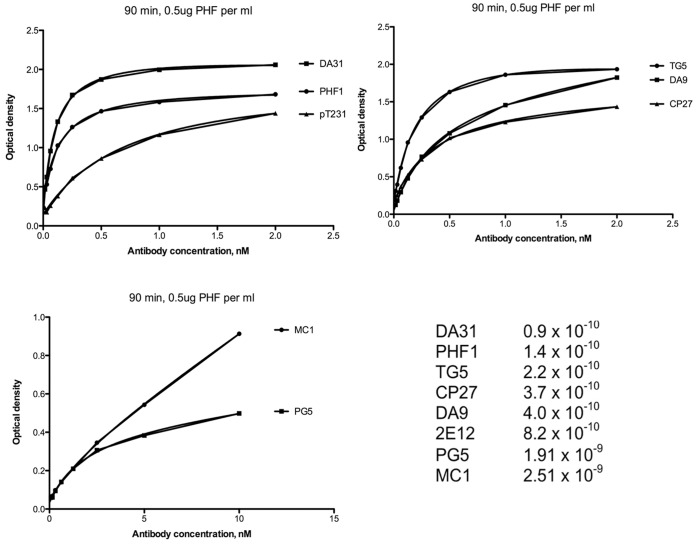
Relative affinities of tau monoclonal antibodies. PHF-tau (0.5 µg/ml) purified from Alzheimer brain was used in ELISA in order to determine the relative binding abilities of a battery of tau monoclonal antibodies. In this assay, DA31 and PHF1 have much higher relative affinities for PHF-tau than does MC1.

MC1 (IgG1) is a well-characterized antibody that recognizes only tau in a pathological conformation, while DA31 (IgG1) is a high affinity tau sequence antibody that recognizes all forms of tau. PHF1 (IgG1) belongs to the group of antibodies directed to phospho-epitopes present on both normal adult brain tau and on PHF-tau, pSer396/404. Measurement of relative affinities of these antibodies is difficult, due to variation in the source of the tau. We have used PHF-tau (0.5 µg/ml) purified from the Alzheimer’s brain in a simple ELISA assay to determine the relative binding abilities of several of our monoclonal antibodies. In this type of assay, DA31 and PHF1 have been shown to have much higher relative affinities for PHF-tau than MC1 (Figure 1A).

We have determined the serum half-life of the antibodies following IP injection, and found that it is greater than 14 days (data not shown). Injections of 250 µg of Ab weekly allowed us the maintenance of a fairly constant serum concentration of 50–75 µg/ml over the duration of the therapy (4 months). Serum antibody concentrations were measured after 4 weeks of treatment and at termination, using PHF-tau coated plates ELISA in order to ensure measurement of active antibody.

### Brain Sample Preparation

At termination of the treatment, mice were sacrificed by isoflurane overdose, decapitated and processed as previously described [21]. Briefly, the brain was removed and divided at the midline so that just one half of brain was dissected for biochemical analysis. Forebrain and hindbrain were homogenized separately using an appropriate volume of homogenizing buffer, a solution of Tris-buffered saline (TBS), pH 7.4, containing 10 mM NaF, 1 mM Na_3_VO_4_ and 2 mM EGTA, plus a complete Mini protease inhibitor cocktail [2]. Samples of brain were stored at −80°C and used for separate measurement of total tau and insoluble tau.

Total tau was measured using heat-stable fractions from brain, as this procedure very effectively removes any mouse IgG (endogenous or exogenous) and simplifies the interpretation of the biochemical results [22]–[25]. Heat stable fractions were prepared by adding 5% ß-Mercaptoethanol and 200 mM NaCl to brain homogenates. Samples were then heated at 100°C for 10′, and cooled at 4°C for 30′. After centrifuging at 13000 g in a table-top microcentrifuge at 4°C for 15′, supernatants were collected and 5X sample buffer (Tris-buffered saline, pH 6.8 containing 4% SDS, 2% beta-mercaptoethanol, 40% glycerol and 0.1% bromophenol blue) was added.

To obtain insoluble tau preparation [26], 500 µl of homogenate were thawed and spun at 6000 g for 10′ at 4°C. The collected supernatant was centrifuged at 200000 g for 30′ at 25°C. The pellet was resuspended in 450 µl of homogenizing buffer and centrifuged again at 200000 g for 30′ at 25°C. The final pellet was resuspended in 200 µl of 1X sample buffer, and heated at 100°C for 10 minutes to efficiently dissociate the insoluble tau.

### Tau Sandwich ELISA

Tau sandwich ELISA was performed as already published [21]. 96-well plates were respectively coated with DA31, CP13 or RZ3 at a final concentration of 6 µg/ml in coating buffer, for at least 48 h at 4°C. After washing 3X in wash buffer, the plates were blocked for 1 h at room temperature using StartingBlock (Thermoscientific) to avoid non-specific binding. Each plate was then washed 5X and 50 µl of the appropriate sample was added to the wells. Concurrently, 50 µl of DA9-HRP detection antibody was added to the samples and tapped to combine. Plates were incubated O/N shaking at 4°C and then washed 9X in wash buffer. 1-Step ULTRA TMB-ELISA (Thermoscientific) was added for 30′ at room temperature before stopping the reaction with 2 M H_2_SO_4_. Plates were read with an Infinite m200 plate reader (Tecan) at 450 nm.

### Tau Monoantibody ELISA

Tau Monoantibody sandwich ELISA was performed as already published [26]. Plates were coated with DA9 (total tau, aa 102–140) at a concentration of 6 µg/ml in coating buffer for at least 48 hours at 4°C. Plates were washed 3× in wash buffer then blocked with StartingBlock (Thermo Scientific). 50 µl of total lysates and PHF-tau, as a standard, were incubated overnight at 4°C. After washing 5×, the total tau detection antibody DA9-HRP was added and incubated for 2 hours at room temperature. The following steps are same as described above.

### Immunocytochemistry

After decapitation, half of brain was fixed overnight in 4% paraformaldehyde at 4°C. Serial sections were cut from the fixed brain half on a vibratome, conserved in TBS (50 mM Tris, 150 mM NaCl, pH 7.6)/0.02% NaN_3_, and stained on multiwell plates with a panel of tau antibodies, using a standardized protocol [22]–[25], [27]–[29], Endogenous peroxidases were quenched with 3% H2O2/0.25% Triton X-100/TBS for 30′. Non-specific binding was blocked with 5% Milk-TBS for 1 hour at room temperature. Primary antibodies were used as followed: anti Tau antibodies CP13 (1/5000), RZ3 (1/500) all diluted in 5% Milk-TBS, and left overnight at 4°C, shaking. Biotin-conjugated secondary antibodies directed against the specific isotypes were diluted 1/1000 in 20% Superblock, left for 2 hours at room temperature, and lately Streptavidin-HRP was incubated for 1 hour. Staining was visualized by 3,3′-Diaminobenzidine. Each step was followed by 5×5′ washes in TBS.

### Statistical Analysis

GraphPad Prism was used for statistical analysis of differences between treated and untreated mice, using ANOVA to test for significant differences between groups. ImageJ software was used to analyze immunociytochemistry staining differences between groups.

## Results

### MC1 Significantly Reduces Total Tau and Insoluble Tau in Forebrains of P301L Mice Treated from 3 to 7 Months of Age, Whereas DA31 does not

In the initial study, P301L mice received weekly intraperitoneal injections of either DA31, MC1 or saline (10 mg/kg, n = 15), beginning at 12 weeks of age. The half-life of the antibodies in the serum of these mice was more than 14 days. A total of 17 injections were performed and the animals were sacrificed at 29 weeks.

**Figure 2:**
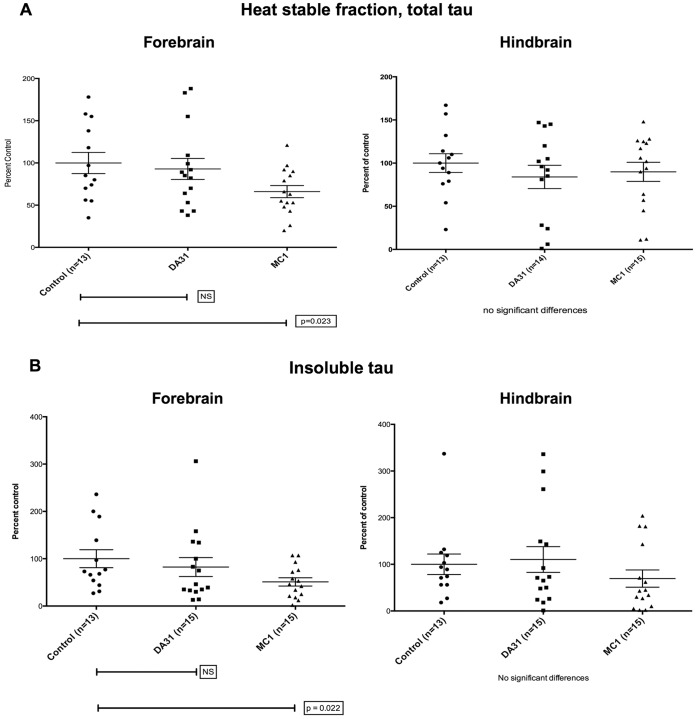
P301L mice (n = 13–15 per group) immunized with MC1 or DA31, 3 to 7 months of age. a) MC1-treated mice display a significant reductions (p = 0.023) in forebrain total tau, while DA31-treated mice do not differ from controls. b) When injecting MC1 the mice exhibit a significant decrease (p = 0.022) in forebrain insoluble pathological tau, and again DA31 fails in exerting any beneficial effect. No significant differences were detected when analyzing the hindbrains of treated versus aged-matched controls (a–b).

The antibody treatment was well tolerated, with only two mouse deaths occurring over 4 months, both in the saline group. As expected, all measures of tau pathology in these mice showed a high degree of variability. However, MC1-treated mice showed significant reductions (p<0.05) in both forebrain total tau (Figure 2A) and forebrain insoluble pathological tau (Figure 2B). Measurement of amounts of total and insoluble tau in hindbrain fractions did not reveal any significant differences between groups (Figure 2A–B). DA31-treated mice did not differ from controls on any biochemical measure (Figure 2A–B).

**Figure 3:**
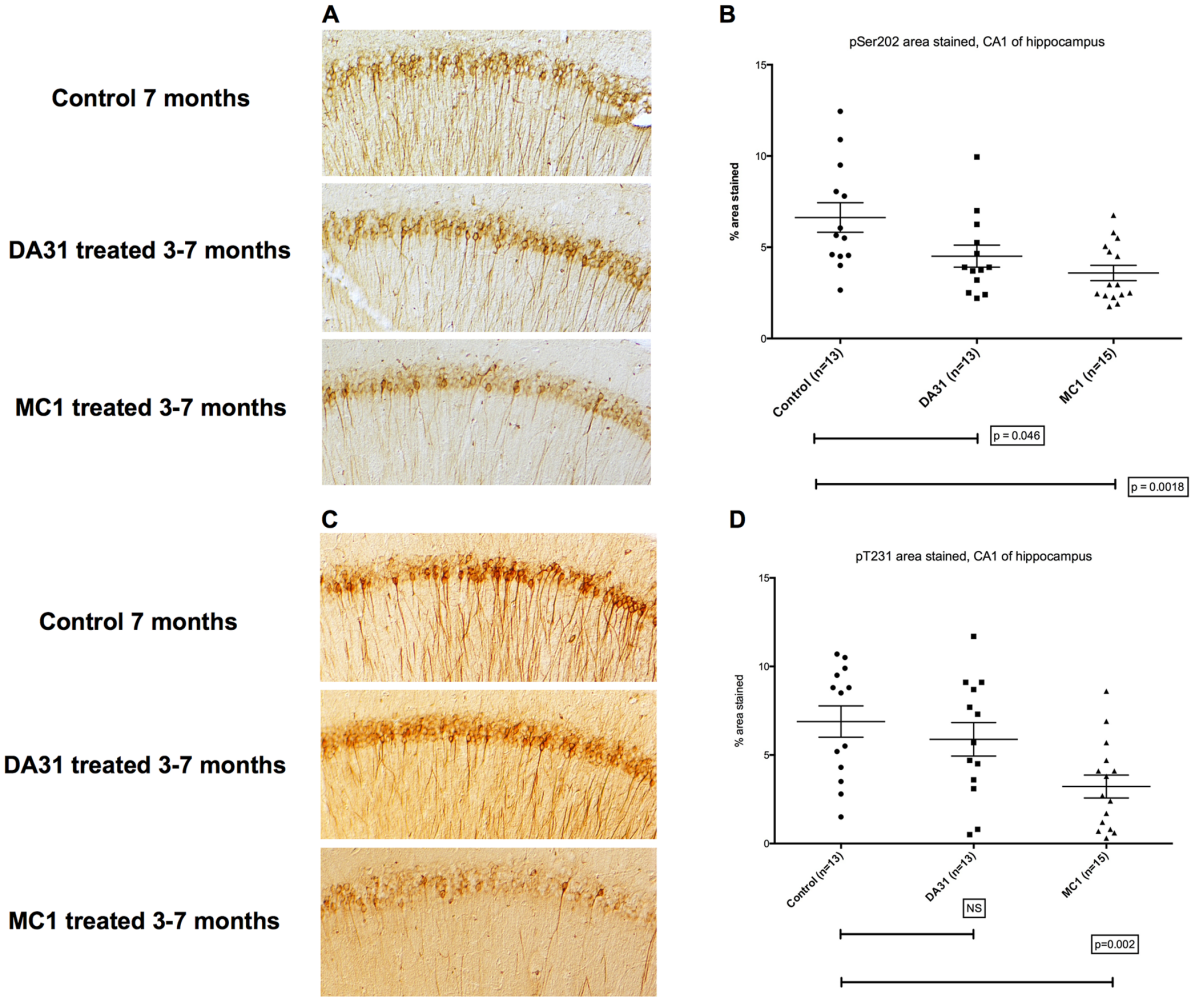
Representative CP13 and RZ3 immunohistochemistry of immunized P301L mice, 3 to 7 months of age. The CA1 hippocampal region of either MC1 or DA31 treated mice was stained with two different phospho-tau antibodies. A–b) CP13 (pSer202) staining is significantly reduced (p = 0.0018) after injecting MC1 or DA31 (p = 0.046). c-d) RZ3 (pThr231) immunoreactivity displays a significant decrease (p = 0.002) in mice treated with MC1, while DA31 does not show any obvious changes. Results are expressed as percent of area stained.

Hippocampal pathology visualized using antibodies to pS202 (CP13) (Figure 3A–B) and pT231 (RZ3) (Figure 3C–D) were both significantly lower (p<0.01) in MC1 treated mice. In DA31-treated mice, there was a significant reduction in pS202 staining (p<0.05), but not in staining for pT231 (Figure 3B–D).

There was no evidence of increased microglia or astrocyte activation following antibody treatment (data not shown).

### P301L Mice Injected with MC1 from 7 to 10 Months of Age Show Decreased Staining of the Hippocampal CA1 Region, and Reduced Phosphorylation of Forebrain Insoluble Tau

**Figure 4:**
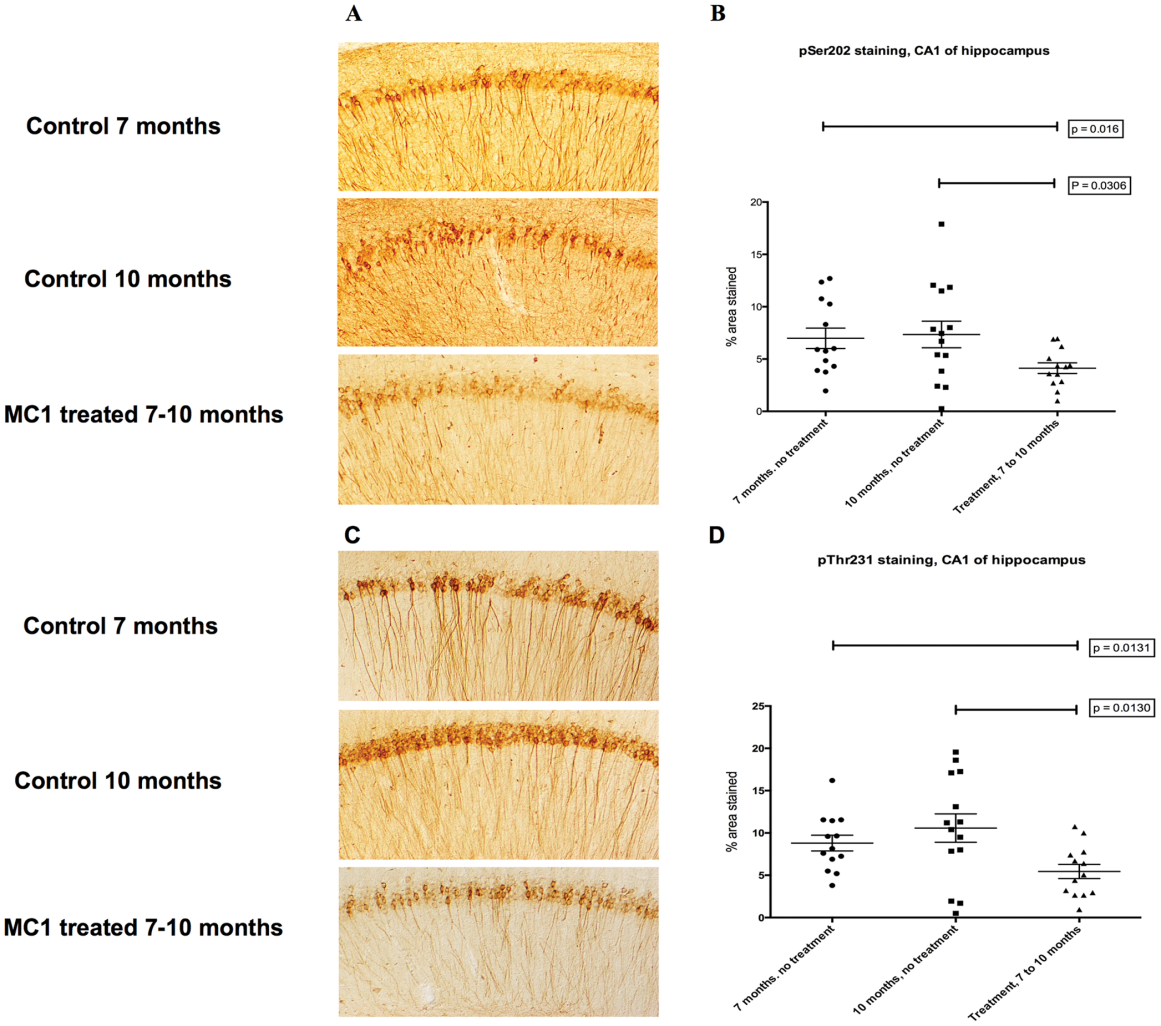
Representative CP13 and RZ3 immunohistochemistry of immunized P301L mice, 7 to 10 months of age. The CA1 hippocampal region of the MC1 treated mice was stained with two different phospho-tau antibodies. a–b) CP13 (pSer202) staining is significantly reduced after injecting MC1, both when comparing 7 and 10 months old controls mice with MC1 treated animals from 7 to 10 months of age (p = 0.016 and p = 0.036 respectively). c–d) RZ3 (pThr231) immunoreactivity displays a significant decrease in mice treated with MC1 from 7 to 10 months of age (p = 0.013). Results are expressed as percent of area stained.

**Figure 5:**
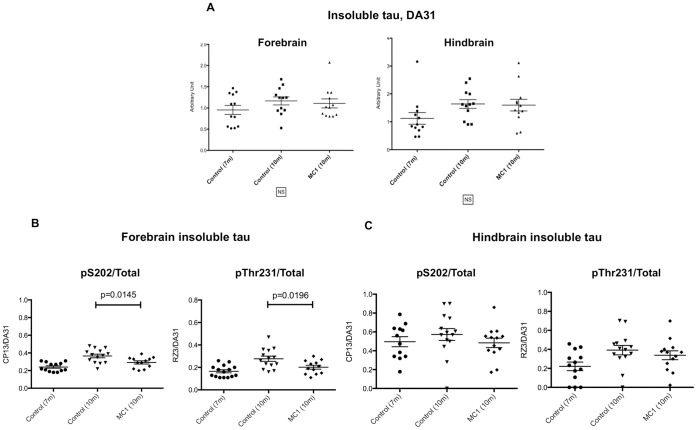
P301L mice (n = 13–15 per group) immunized with MC1, 7 to 10 months of age. a) Insoluble tau analysis, using the DA31-DA9hrp ELISA, does not show any obvious change both in forebrain and hindbrain of treated mice. b) In forebrain, the ratios of insoluble pS202 and pThr231 over total tau are significantly reduced (p = 0.0145, p = 0.0196). The hindbrain analysis does not show any effect.

All the published studies of tau immunotherapy to date have examined the effects of treatment before extensive pathology had developed. Hence, a second series of mice were treated with MC1 to determine if this antibody was also effective when given later in the course of the disease. P301L mice aged 7 months were injected with MC1 (250 µg) once weekly for 14 weeks. There were two control groups: 13 P301L mice were killed at 7 months of age (zero time for this study - no treatment), and 13 P301L mice were injected with saline once weekly for 14 weeks. The study was terminated after 14 weeks because 8 of 13 saline-injected mice showed signs of hind limb weakness. Only 1 of the MC1-injected mice presented such signs, and the study was terminated with the mice approximately 10.5 months old. Immunohistochemical analysis showed reduced staining for pS202 and pT231 in the CA1 region of the hippocampus following MC1 treatment (Figure 4A–C). Areas stained were significantly lower than in saline injected mice and compared to control mice killed at 7 months of age (Figure 4B–D). While MC1 appeared to lower the burden of tau pathology in the hippocampus, biochemical analysis of heat-stable tau did not reveal any significant differences between treated groups (data not shown). In the forebrain, the ratios of insoluble pS202 and pThr231 over total tau were significantly reduced (p = 0.0145, p = 0.0196) (Figure 5B), correlating well with the hippocampal staining. MC1 treatment did not result in any reduction in insoluble tau levels in either forebrain or hindbrain when initiated at 7 months of age (Figure 5A).

### The Survival Rate of MC1 or PHF1 Injected Mice, from 6 to 14 Months of Age, does not Increase Compared to Control Animals

**Figure 6:**
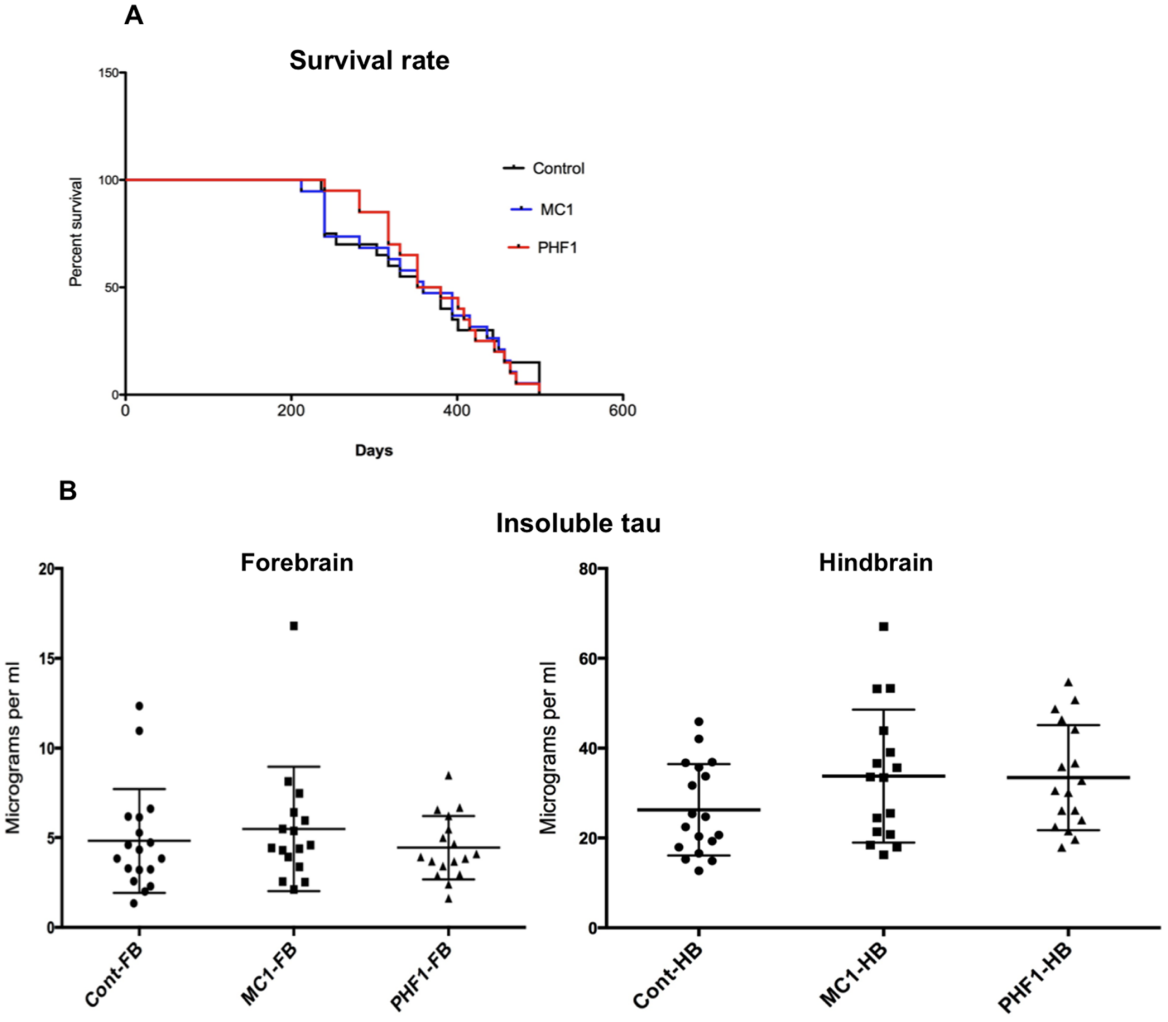
Survival experiment. a) P301L mice were treated with MC1, PHF1 or saline from 7 months of age (200 days): no difference in the survival rate is observed between the animal cohorts. b) The analysis of forebrain and hindbrain insoluble tau does not show any difference between groups, correlating well with the survival rate data.

Since MC1-treated mice showed significant reduction of tau pathology, a third series of mice were injected in order to evaluate possible effects on survival. We included in this study a cohort of animal treated with PHF1, an antibody recognizing the pThr396-404 epitope present on both normal adult brain tau and PHF-tau. PHF1 has already been demonstrated to be efficacious at reducing pathological tau by another group [15]. During the study several animals died or were sacrificed due to severe motor dysfunction. The experiment was terminated approximately at 14 months. We were not able to detect any difference in the survival rate (Figure 6A). Furthermore, the levels of insoluble tau from forebrain and hindbrain, analyzed using the monoantibody ELISA [26], did not appear to be different in treated or control mice (Figure 6B).

### IgG1 do not Appear to Enter within the Neurons

**Figure 7:**
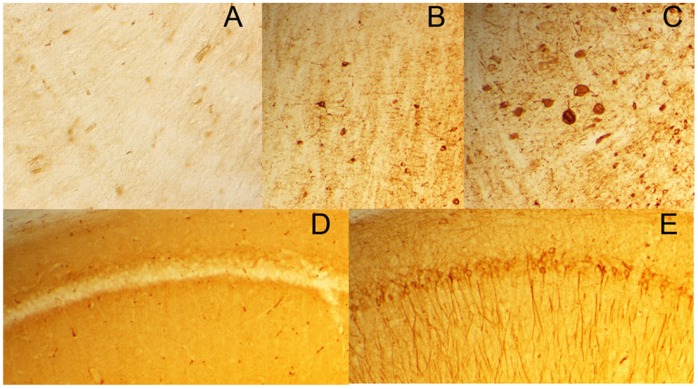
IgG do not enter into neuronal cell bodies. A) Goat anti-mouse IgG1 followed by Streptavidin-HRP staining: some immunoreactivity from the blood vessels, but no detectable staining of neurons. B–C) PG5 (pSer409) staining of brain stem of P301L mice: neuronal staining is readily detectable when present. D) Biotinylated-secondary antibody followed by Streptavidin-HRP staining: no neuronal reactivity is seen. Blood vessels staining is detectable in the hippocampus of the mice. E) The same mouse does show any tau pathology (CP13) in hippocampal pyramidal cells.

P301L mice treated for 4 months with MC1 or DA31 have been carefully examined for the presence of antibodies within neurons. The secondary antibody detection system we use for immunocytochemistry involves incubation of tissue sections with goat anti-mouse IgG1-biotin, and then Streptavidin-HRP. This is an extremely sensitive and specific means of detecting antibodies bound to tissue sections, and ought to be able to detect immunoglobulins in sections when no primary antibody is used. We have examined sections from every P301L mouse injected with MC1 or DA31 but no evidence of neuronal IgG1 staining has been detected, although even small numbers of stained neurons in brain stem are not hard to detect when a tau primary antibody is used (Figure 7A–E).

## Discussion

In the present study, we have tested the hypothesis that specificity of monoclonal antibodies directed to tau is an important determinant of efficacy in passive immunotherapy. MC1 recognizes an AD specific tau conformational epitope, while DA31 and PHF1 are high affinity antibodies detecting pan-tau or p-tau in both normal and Alzheimer’s brain. As shown in Figure 1a, the monoclonal antibodies MC1 and DA31/PHF1 stand on the polar opposites in terms of affinity for PHF-tau, and so does their specificity for pathological tau. Weekly intraperitoneal injections (250 µg) of MC1, DA31 or PHF1 were administered to different cohorts of mice carrying the P301L tau mutation responsible for frontotemporal dementia in humans. We performed three distinct studies: a) 3 months old mice were injected until the age of 7 months with MC1 or DA31; b) 7 months old mice were treated until the age of 10.5–11 months with MC1; c) 6 months old mice were administered MC1 or PHF1 to detect their survival rate until 14 months of age maximum. A mock-treated group of age-matched mice was included in each study and injected with saline. As stated previously, we have been reluctant to use a “non-specific” mouse IgG as a control, because of reports of efficacy of intravenous IgG in the treatment of Alzheimer’s disease [30]. The selection of a particular mouse monoclonal antibody as a “non-specific” IgG seems inappropriate, given that it is currently unknown if there are “non specific” effects on the human disease; in that particular experimental model we would be undervaluing the effect of tau antibodies in immunotherapy.

The first experiment of the series was performed on 3 months old P301L mice in order to test if early treatment with tau monoclonal antibodies could “prevent” tau pathology. Biochemical analysis of total and insoluble tau together with hippocampal staining showed a striking superiority of MC1 at reducing the tau burden compared to DA31. Since we were not able to detect any antibody within the neurons, and assuming that tau is physiologically released to the extracellular space, we can support the notion that the mechanism by which these antibodies work is by targeting extracellular PHF-tau. However, why a conformational antibody would work better at reducing tau pathology compared to a pan-tau antibody is still unclear. Our thought is that MC1 specificity in targeting PHF-tau allows this antibody to find pathological tau even if present at a very low concentrations. On the contrary, DA31 targets every form of extracellular tau without any specificity for PHF-tau. Considering that in the early stages of Alzheimer’s disease the amount of “normal” extracellular tau is probably higher overall, the inability to discriminate between PHF-tau and “normal” tau could result in losing therapeutic effect.

Due to the effectiveness of MC1 in reducing the pathology when used early in the disease, the second study focused on “clearing” preexisting tau pathology: MC1 was injected in older P301L mice, from 7 to almost 11 months of age. The hippocampal CA1 staining showed significant reduction of pathological tau aggregates in the injected mice, in parallel with biochemical data showing decreased phosphorylated insoluble tau. We were not able to detect any effect analyzing total tau or insoluble tau levels. Furthermore, 8 of 13 saline-injected mice showed signs of hind limb weakness whereas only 1 of the MC1-injected mice presented such manifestation. We conclude that MC1 is more effective at prevention of development of tau pathology than at reducing pre-existing pathology, although some benefits were apparent.

With this in mind, the third study was carried out to determine if antibody therapy was effective at prolonging the life of the P301L mice. Six months old P301L mice were injected with MC1 or PHF1 until severe signs of motor dysfunction occurred. Since DA31 did not exert any beneficial effect in clearing the NFT burden in tau mutant mice, we selected PHF1 as representative of the high affinity tau antibodies to be used in this investigation. PHF1 has already been shown to be efficient at reducing tau pathology when used early in immunotherapy treatments [15]. Mice were killed when exhibiting severe lack of escape extension (clasping) together with weakness and paralysis, but both MC1 and PHF1 treated mice did not differ from the saline injected cohort in terms of survival time or amount of insoluble tau. The reason why we were not able to increase the vitality of the immunized mice is unknown. It could be speculated that an earlier intervention (3 months of age) could have been more effective in improving the survival rate of the injected mice, since both MC1 and PHF1 were efficacious in reducing tau pathology in previous studies.

In summary, this work strongly supports the idea that passive immunotherapy in mutant tau mice can be efficacious in reducing the development of tau pathology. The conformational antibody MC1 appears overall to be superior at reducing tau pathology in P301L mice compared to the high affinity pan-tau antibody DA31: this may shed light on the idea that specificity is more important than affinity in therapeutic applications. Unfortunately, the vitality of the injected mice did not benefit from tau immunotherapy, independently from specificity or affinity of the antibody selected. It is still far from clear if mice with normal human tau transgenes would benefit from treatment with these antibodies. It is also extremely important to establish what characteristics define an effective antibody in different mouse models. Further exploration of possible mechanism of efficacy using both in vivo and vitro systems is clearly important. Considering the potential future translation to human therapeutic trial, it is obvious that a great deal of work remains to be done before suggesting that treatment of Alzheimer’s disease with humanized tau antibodies should proceed.
